# Nurse Practitioners’ Experiences of Polypharmacy in Community-Dwelling Older Adults

**DOI:** 10.1093/geroni/igaa057.670

**Published:** 2020-12-16

**Authors:** Christine Pariseault, Nancy Sharts-Hopko, Elizabeth Blunt

**Affiliations:** Villanova University, Villanova, Pennsylvania, United States

## Abstract

Numerous studies exist that define polypharmacy and its impact on health. Additionally, the literature is rich in studies documenting the benefits of care provided by nurse practitioners. A gap in research exists at the intersection of the value of nurse practitioners in caring for older adults and their management of polypharmacy. Coinciding with a growth of America’s older adult population and the need for adequate care, the purpose of this study was to explore the experiences of nurse practitioners caring for older adults experiencing polypharmacy. A qualitative descriptive study was conducted using a purposive sampling of nurse practitioners who care for older adults. Interviews were conducted and data was analyzed for themes. Four themes emerged: defining polypharmacy, communicating and collaborating, clinical judgement of nurse practitioners in relation to polypharmacy, and medication issues of older adults. Major themes emerged that depict the complexity of medication management in older adults as well as the important role of NPs in providing care to older adults. The significance of the study findings to future practice includes improving communication and collaboration of prescribing health care providers, better identification and management of polypharmacy, and improving the health care delivered to older adults. Safe and effective prescribing for older adults requires NPs consider the unique needs of each older adult while utilizing technology to support collaboration and decision making.

